# Neuregulin1 Attenuates H_2_O_2_-Induced Reductions in EAAC1 Protein Levels and Reduces H_2_O_2_-Induced Oxidative Stress

**DOI:** 10.1007/s12640-018-9965-4

**Published:** 2018-10-17

**Authors:** Jun-Ho Lee, Ji-Young Yoo, Han-byeol Kim, Hong-Il Yoo, Dae-Yong Song, Sun Seek Min, Tai-Kyoung Baik, Ran-Sook Woo

**Affiliations:** 1 Department of Emergency Medical Technology, Daejeon University, Daejeon, 34520 Republic of Korea; 2 Department of Anatomy and Neuroscience, College of Medicine, Eulji University, 143-5, Yongdu-Dong, Jung-Gu, Daejeon, 34824 Republic of Korea; 3 Department of Physiology and Biophysics, College of Medicine, Eulji University, Daejeon, 34824 Republic of Korea

**Keywords:** H_2_O_2_, Neuregulin 1, EAAC1, Reactive oxygen species, Superoxide dismutase, Glutathione peroxidase

## Abstract

Neuregulin 1 (NRG1) exhibits potent neuroprotective properties. The aim of the present study was to investigate the antioxidative effects and underlying mechanisms of NRG1 against H_2_O_2_-induced oxidative stress in primary rat cortical neurons. The expression level of the excitatory amino acid carrier 1 (EAAC1) protein was measured by Western blotting and immunocytochemistry. The levels of lactate dehydrogenase (LDH) release, reactive oxygen species (ROS) generation, superoxide dismutase (SOD) activity, GPx activity, and mitochondrial membrane potential (∆ψm) were determined to examine cell death and the antioxidant properties of NRG1 in primary rat cortical neurons. H_2_O_2_ reduced the expression of EAAC1 in a dose-dependent manner. We found that pretreatment with NRG1 attenuated the H_2_O_2_-induced reduction in EAAC1 expression. Moreover, NRG1 reduced the cell death and oxidative stress induced by H_2_O_2_. In addition, NRG1 attenuated H_2_O_2_-induced reductions in antioxidant enzyme activity and ∆ψm. Our data indicate a role for NRG1 in protecting against oxidative stress via the regulation of EAAC1. These observations may provide novel insights into the mechanisms of NRG1 activity during oxidative stress and may reveal new therapeutic targets for regulating the oxidative stress associated with various neurological diseases.

## Introduction

Evidence of oxidative stress in the brain has been reported for aging and a variety of neurological and neurodegenerative diseases. Oxidative stress is characterized by excessive production of reactive oxygen species (ROS) and a lack of ROS clearance and is known to cause lipid peroxidation, excitotoxicity, and neuronal damage (Dringen et al. 2000; Gandhi and Abramov 2012; Valko et al. 2007).

Excitatory amino acid transporters (EAATs) regulate glutamatergic signaling by taking up glutamate from synaptic clefts into the cells. Among the five EAAT isoforms, EAAT1-3 is the most widely expressed in the brain. EAAT1 (glutamate-aspartate transporter, GLAST) and EAAT2 (glutamate transporter-1, GLT1) are expressed in glial cells and are mainly involved in synaptic glutamate clearance, while EAAT3 (excitatory amino acid carrier 1, EAAC1) is predominantly expressed in neurons but is also expressed in other types of cells. EAATs are believed to be important factors for the prevention of damage due to excitotoxicity (Amara and Fontana 2002). Interestingly, EAAC1 is also involved in cysteine uptake and might mediate most of the influx of this amino acid in neurons (Aoyama and Nakaki 2015). Mature neurons utilize extracellular cysteine for glutathione (GSH) synthesis, while astrocytes utilize cystine, which is formed by the oxidation of two cysteines with a disulfide bond. Intracellular cysteine is the rate-limiting substrate for the synthesis of GSH, the principal cellular antioxidant (Griffith 1999). Therefore, cysteine transport via EAAC1 is considered key for neuronal GSH synthesis. GSH is important for the metabolism of hydrogen peroxide (H_2_O_2_), nitric oxide, and other reactive oxygen species and for the maintenance of reduced thiol groups on proteins (Dringen 2000). Furthermore, EAAC1 expression is altered in pathological conditions such as schizophrenia, hypoxia/ischemia, multiple sclerosis, and epilepsy (Bauer et al. 2008; Bianchi et al. 2014; Crino et al. 2002; Romera et al. 2004).

Neuregulin 1 (NRG1) is a member of a group of growth and differentiation factors that is thought to have important roles in regulating brain development. Several lines of evidence have demonstrated the role of NRG1 in the regulation of synaptic plasticity and neurotransmission (Kwon et al. 2005; Li et al. 2007; Woo et al. 2007). NRG1 protects neurons under various conditions of stress, including ischemia, organophosphate-induced neural injury, and Alzheimer’s disease (Guo et al. 2006; Li et al. 2012; Ryu et al. 2016; Woo et al. 2012). We have previously reported the neuroprotective effects of NRG1 in cell models and in a transgenic mouse model of Alzheimer’s disease (Ryu et al. 2016). Furthermore, we have found that NRG1 controls glutamate uptake by EAAC1 (Yu et al. 2015). These findings suggest that NRG1 could be involved in the regulation of abnormal EAAC1 expression in oxidative stress.

In the present study, we evaluated the effects of NRG1 on H_2_O_2_-induced oxidative stress in primary cortical neurons.

## Materials and Methods

### Reagents and Antibodies

The NRG1 used in this study was a recombinant polypeptide containing the entire EGF domain of the β-type NRG1 from PROSPEC (East Brunswick, NJ, USA). Antibodies were supplied by Millipore Corporation (Chemicon, MA, USA) (EAAT3 (EAAC1), MAB1587), Santa Cruz Biotechnology Inc. (Santa Cruz, CA, USA) (β-actin, sc-47778; HRP-conjugated anti-rabbit IgG, sc-2004; and HRP-conjugated anti-mouse IgG, sc-2005), and Cell Signaling Technology (CST, MA, USA) (EAAC1, #12179). H_2_O_2_ (216763) and Rhodamine 123 (Rh123, 83702) were purchased from Sigma-Aldrich (St. Louis, MO, CA, USA).

### Cell Culture

Primary cortical neurons were cultured as described previously (Woo et al. 2007). Briefly, the cerebral cortex was removed from Sprague-Dawley rat embryos (E18) and dissociated by gentle trituration in PBS (Gibco, Carlsbad, CA, USA). Cells were seeded on poly-l-lysine-coated 6-well plates and cultured in Neurobasal media (Gibco). Experiments were performed 14 days after seeding (DIV14).

### Lactate Dehydrogenase Release Assay

Degrees of cell death were assessed by the activity of lactate dehydrogenase (LDH) released into the culture medium using a Cytotox 96 nonradioactive cytotoxicity assay kit (Promega, Madison, WI) according to the manufacturer’s instructions. The results are expressed as the percentage of maximum LDH release obtained upon complete cell lysis.

### Measurement of Reactive Oxygen Species (ROS) Generation

ROS generation was measured in primary cortical neurons using the dye 2′,7′-dichlorodihydrofluorescein diacetate (DCFH-DA; Invitrogen, CA, USA). Cells were washed twice with HEPES-buffered saline and incubated for 1 h in the dark in HEPES-buffered saline that contained DCFH-DA (200 μM). Esterase was used to cleave the acetoxymethyl group of DCFH-DA, which was oxidized to dichlorofluorescein (DCF) in the presence of reactive oxygen species. Intracellular fluorescence was measured using a spectrofluorometer (VICTOR2, PerkinElmer, USA) at an emission wavelength of 529 nm and an excitation wavelength of 504 nm (Baik et al. 2016).

### Superoxide Dismutase (SOD) Activity Assay

The SOD activity was assessed using a commercially available kit (Cayman Chemical Company, MI, USA) following the manufacturer’s instructions. Briefly, collected cells were homogenized with lysis buffer (20 mM HEPES (pH 7.2), 1 mM EGTA, 210 mM mannitol, and 70 mM sucrose) and then centrifuged at 1500 ×*g* for 5 min at 4 °C. The cell suspension was then centrifuged at 1500 ×*g* for 5 min at 4 °C. The assay sample was mixed with 190 μl of the diluted radical detector. The reaction was initiated by adding 20 μl of diluted xanthine oxidase. The plate was incubated on a shaker for 20 min at room temperature. The absorbance was read at 450 nm using a VICTOR X3 Multilabel plate reader (PerkinElmer, Shelton, USA) (Baik et al. 2016).

### Glutathione Peroxidase (GPx) Activity Assay

The activity of GPx was measured using a commercially available kit (Glutathione Peroxidase Activity Assay Kit, # K762–100, BioVision Research, CA, USA) following manufacturer’s instructions. Cells were collected and homogenized with lysis buffer (20 mM HEPES (pH 7.2), 1 mM EGTA, 210 mM mannitol, and 70 mM sucrose). The cell suspension was then centrifuged at 10,000 ×*g* for 15 min at 4 °C. We then added 50 μl of supernatant and 50 μl of assay buffer to the wells. We initiated the reaction by adding 40 μl of the reaction mixture to each sample and incubating for 15 min to deplete all GSSG in samples. Then, 10 μl of cumene hydroperoxide solution was added to start the GPx reaction, and the samples were incubated for 5 min at room temperature. A VICTOR X3 Multilabel plate reader (PerkinElmer, Shelton, USA) was then used to read the absorbance at 340 nm.

### Determination of Mitochondrial Membrane Potential (∆ψm)

The potentiometric probe Rhodamine 123 (Rh123) was used to assess the perturbations in ∆ψm. Rh123 is a fluorescence probe that selectively enters into the mitochondria with an intact membrane potential. The fluorescence intensity of mitochondria quantitatively decreases in response to dissipation of ∆ψm. Cells were carefully washed with PBS, and Rh123 (final concentration; 2.5 μg/ml in 5 mM HEPES-buffered saline) was added to the cells. Cells were incubated at 37 °C in the dark for 1 h. The supernatants were discarded and cells were carefully rinsed with PBS before addition of 200 μl per well of fresh HEPES-buffered saline. Fluorescence was directly measured in each well at an excitation wavelength of 485 ± 20 nm and an emission wavelength of 530 ± 25 nm with a spectrofluorometer (Victor 2, Perkin Elmer, USA) (Ryu et al. 2012).

### Immunostaining

Immunostaining of rat cortical neurons (E18, DIV14) was performed as previously described (Woo et al. 2007). Briefly, neurons were fixed with 4% paraformaldehyde and 4% sucrose in PBS for 20 min. The cells were permeabilized by incubation in PBS containing 1% BSA and 0.1% Triton X-100 for 30 min at room temperature. After washing, cells were incubated in buffer containing antibodies against EAAC1 (1:100) at 4 °C overnight and were then incubated with FITC-conjugated AffiniPure goat anti-mouse IgG (Jackson ImmunoResearch Laboratories, Inc., 1:200) in buffer for 2 h at room temperature. Nuclei were counterstained with Hoechst (10 μM in PBS) for 30 min. Stained cells were mounted in VECTASHIELD (Vector Laboratories) and observed under a Zeiss LSM 510 META laser scanning microscope (Carl Zeiss, Germany).

### Western Blotting

Western blotting was performed as previously described (Yu et al. 2015). Samples were resolved using SDS-PAGE and were then transferred to a nitrocellulose membrane, which was blocked with TBS that contained 5% BSA and 0.05% Tween 20 for 1 h. The membrane was incubated with anti-EAAC1 (mouse, 1:1000, Millipore Corporation or rabbit, 1:1000, Cell Signaling Technology) and anti-β-actin (mouse, 1:5000 or rabbit, 1:5000, Santa Cruz Biotechnology) antibodies at 4 °C overnight. After washing, blots were developed with horseradish peroxidase-conjugated secondary antibodies and enhanced using a chemiluminescence system (Amersham Pharmacia, CA, USA).

### Statistical Analysis

Data are presented as the mean ± SEM of three or more independent experiments. For multiple group comparisons, statistical analyses were performed using one-way analysis of variance (ANOVA) followed by Bonferroni’s post-hoc test. Student’s paired *t* test was used for comparisons of the means between two groups of cells in a single experiment. Values of *P* < 0.05 were considered significant.

## Results

### H_2_O_2_ Reduced EAAC1 Protein Levels

**Fig. 1:**
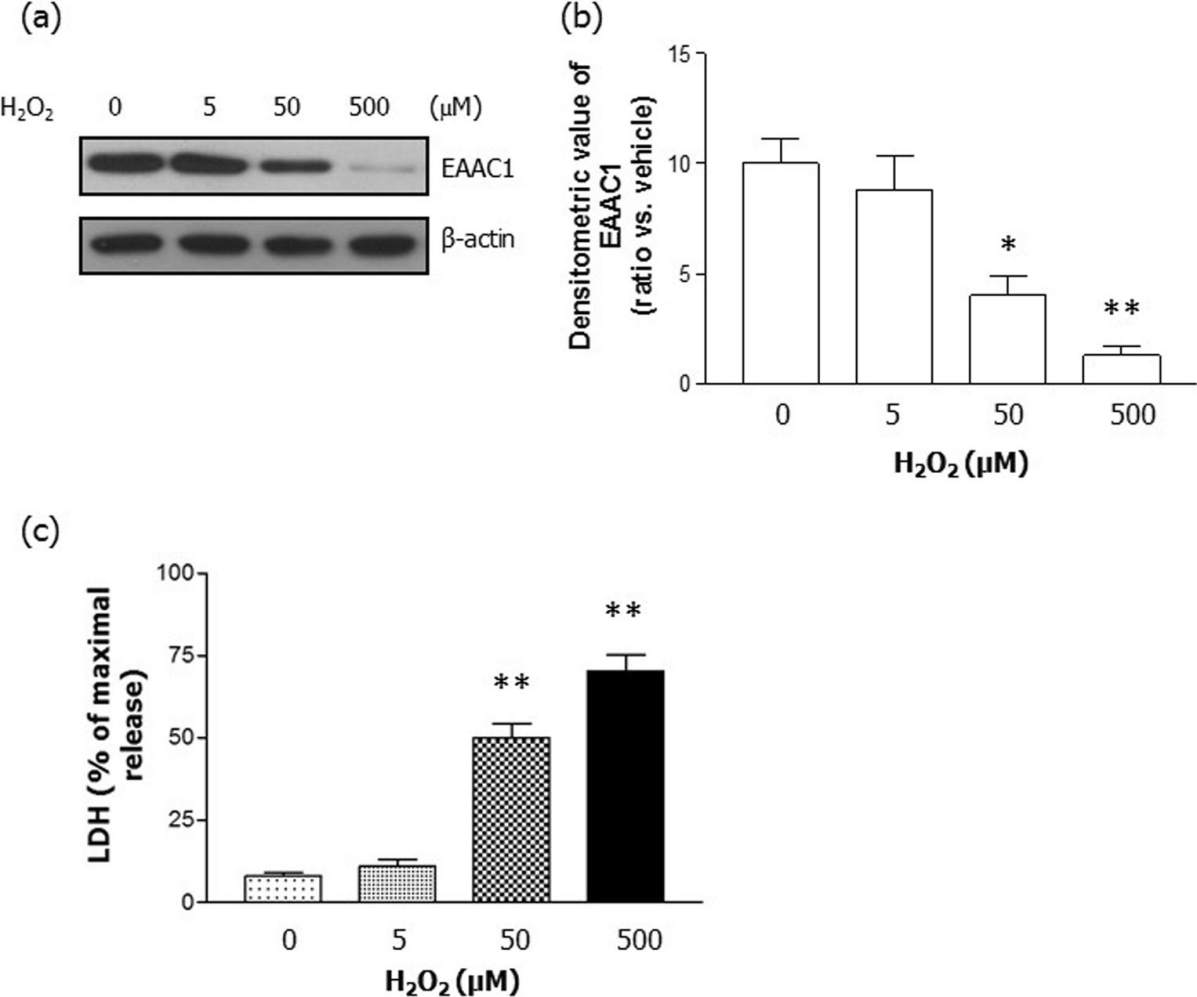
H_2_O_2_ decreased EAAC1 protein expression. **a** Rat primary cortical neurons were treated with varying concentrations of H_2_O_2_ (0, 5, 50, and 500 μM) for 24 h, which resulted in dose-dependent decreases in EAAC1 expression. **b** Quantification of the data in A. Densitometry values are shown as ratios relative to the values of the control group; *n* = 6, *P < 0.05, **P < 0.01. **c** H_2_O_2_ increased neuronal cell death in a dose-dependent manner. The degree of cell death was assessed for 24 h after H_2_O_2_ treatment in primary cortical neurons using LDH activity in the medium; *n* = 5, **P < 0.01

EAAC1 is believed to be important for the synthesis of intracellular glutathione and for subsequent protection from oxidative stress. First, we tested whether H_2_O_2_ regulated EAAC1 protein levels in rat primary cortical neurons. We determined that there was a dose-dependent reduction in EAAC1 expression caused by 24 h of H_2_O_2_ (5–500 μM) treatment (Fig. 1). Quantification demonstrated that H_2_O_2_ significantly reduced the expression of EAAC1 (CON, 10.00 ± 1.16; 5 μM H_2_O_2_, 8.83 ± 1.48; 50 μM H_2_O_2_, 4.00 ± 0.87; 500 μM H_2_O_2_, 1.30 ± 0.42; *n* = 6; Fig. 1a, b). Furthermore, we performed an LDH release assay in rat primary cortical neurons. A 24-h treatment with H_2_O_2_ increased LDH release in a concentration-dependent manner (Fig. 1c). Because treatment with 50 μM H_2_O_2_ had reduced the expression of EAAC1 by 2.3- to 2.8-fold and caused a significant increase in LDH release, this concentration was chosen for further studies.

### NRG1 Rescued EAAC1 Protein Expression

**Fig. 2:**
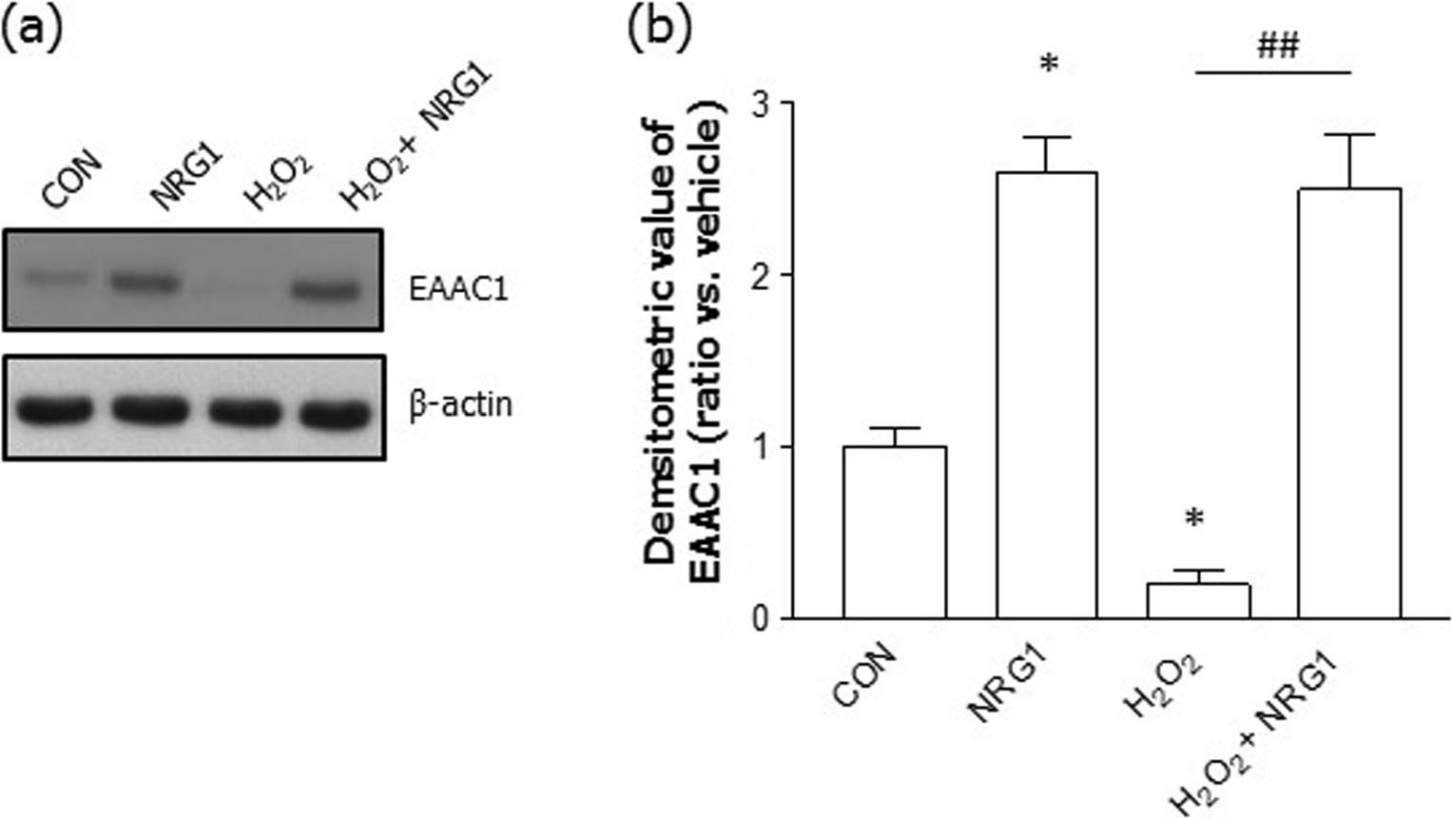
The effects of NRG1 on the protein levels of EAAC1 induced by H_2_O_2_. **a** Representative immunoblots for EAAC1 are shown for primary cortical neurons in the presence or absence of 5 nM NRG1 following treatment with 50 μM H_2_O_2_ for 24 h. **b** Quantitative analysis of the data in A. Treatment with 50 μM H_2_O_2_ significantly decreased the expression of EAAC1. NRG1 attenuated the reduction in EAAC1 expression, as shown by the densitometric values, which are shown as ratios relative to the values of the nontreated control group; *n* = 6, *P < 0.05, **P < 0.01, ^###^*P* < 0.001. Statistical analysis was performed by one-way ANOVA followed by Bonferroni’s post hoc test

To determine whether NRG1 affected the H_2_O_2_-induced reduction in EAAC1 expression, we treated cells with 5 nM NRG1 at DIV14. We then used Western blotting to assay the levels of EAAC1 in rat primary cortical neurons. We confirmed that the treatment of rat primary cortical neurons with 5 nM NRG1 for 24 h significantly upregulated the levels of EAAC1 (CON, 1.00 ± 0.12; NRG1, 2.60 ± 0.21; *n* = 6, *P < 0.05). Moreover, treatment with 50 μM H_2_O_2_ significantly reduced the levels of EAAC1 (0.22 ± 0.09, *n* = 6, **P < 0.01) compared with those in untreated neurons (CON, 1.00 ± 0.12, *n* = 6). Treatment with 5 nM NRG1 for 24 h (from DIV14 to DIV15) attenuated the reduction in EAAC1 expression induced by treatment with H_2_O_2_ (H_2_O_2_, 0.20 ± 0.09, *n* = 6; NRG1, 2.50 ± 0.32, *n* = 6; ^##^*P* < 0.001; Fig. 2a, b).

### NRG1 Alleviated the Decrease in EAAC1 Immunoreactivity in Primary Cortical Neuronal Cells

**Fig. 3:**
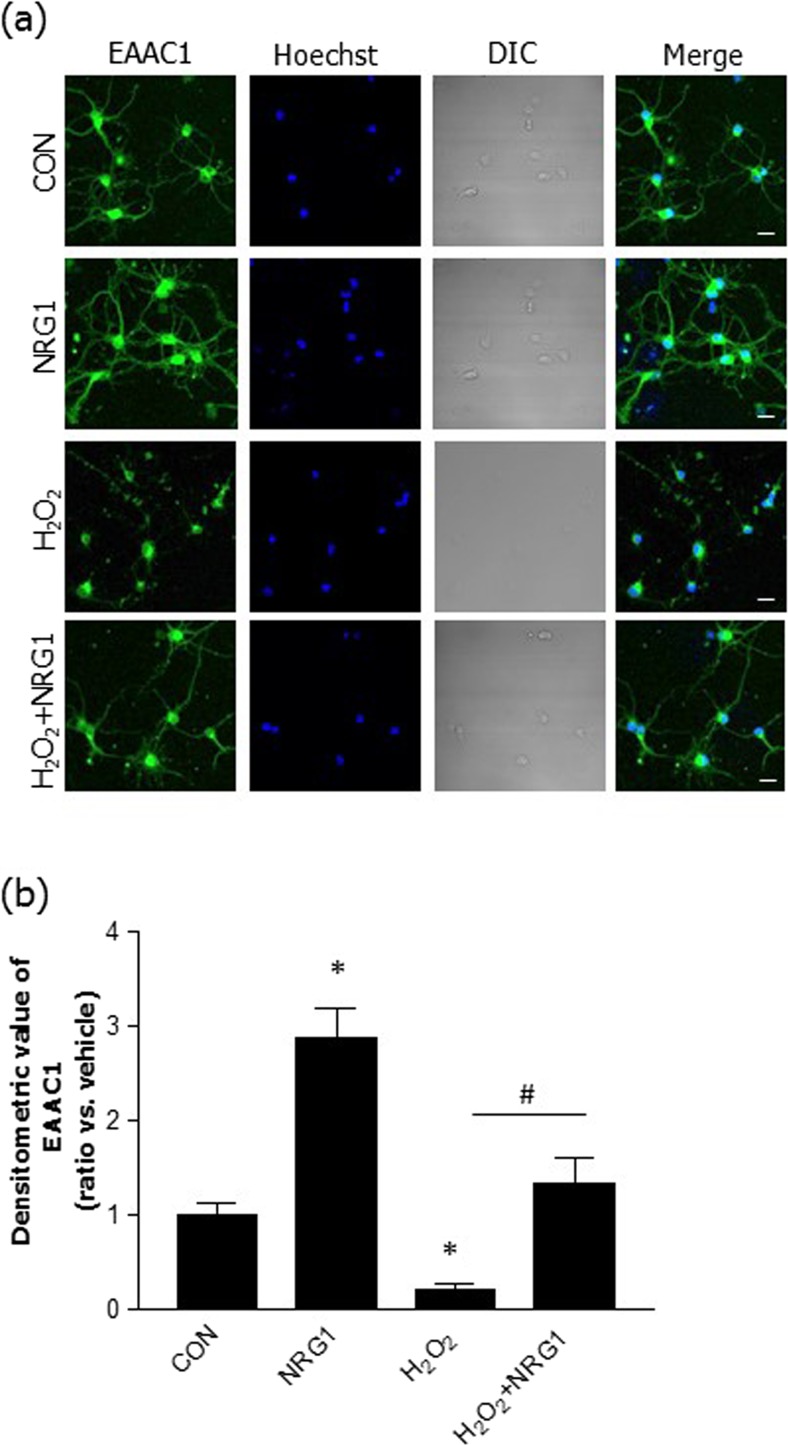
NRG1 attenuated the reduction in EAAC1 protein expression induced by H_2_O_2_ in primary cortical neuronal cells. **a** Immunocytochemical staining with anti-EAAC1 was performed 24 h after 50 μM H_2_O_2_ treatment in the presence or absence of 5 nM NRG1 in primary cortical neuronal cells (at DIV13). The primary cortical neurons were fixed and immunostained with anti-EAAC1 (green), while Hoechst stain (blue) was used as a counterstain. Scale bars, 20 μM. **b** Bar graph summarizing the data from neurons with EAAC1 fluorescence; *n* = 8, *P < 0.05, ^#^*P* < 0.05

We measured the immunoreactivity of EAAC1 via immunocytochemistry in primary cortical neuronal cells. To examine the effects of NRG1 in neurons, primary cortical neuronal cells were pretreated at DIV13 with 5 nM NRG1 and were then treated with 50 μM H_2_O_2_ 15 min later. We then determined the density of immunoreactivity at DIV14 (Fig. 3a). Treatment with 5 nM NRG1 for 24 h significantly upregulated the EAAC1 immunoreactivity in comparison to that of the controls (CON, 1.00 ± 0.09; NRG1, 2.88 ± 0.32, *n* = 8; *P < 0.05). These results are consistent with those of our previous studies demonstrating the effects of NRG1 (Himi et al. 2003). We also confirmed that the treatment of rat primary cortical neuronal cells with 50 μM H_2_O_2_ for 24 h induced a significant decrease in EAAC1 immunoreactivity (0.20 ± 0.06, *n* = 8, *P < 0.05) compared with that of the controls. Treatment with 5 nM for 24 h attenuated the decrease in EAAC1 immunoreactivity induced by treatment with H_2_O_2_ (1.33 ± 0.27, *n* = 8, ^#^*P* < 0.05) (Fig. 3b).

### NRG1 Reduced H_2_O_2_-Induced Cell Stress and Increased Antioxidant Enzymes

**Fig. 4:**
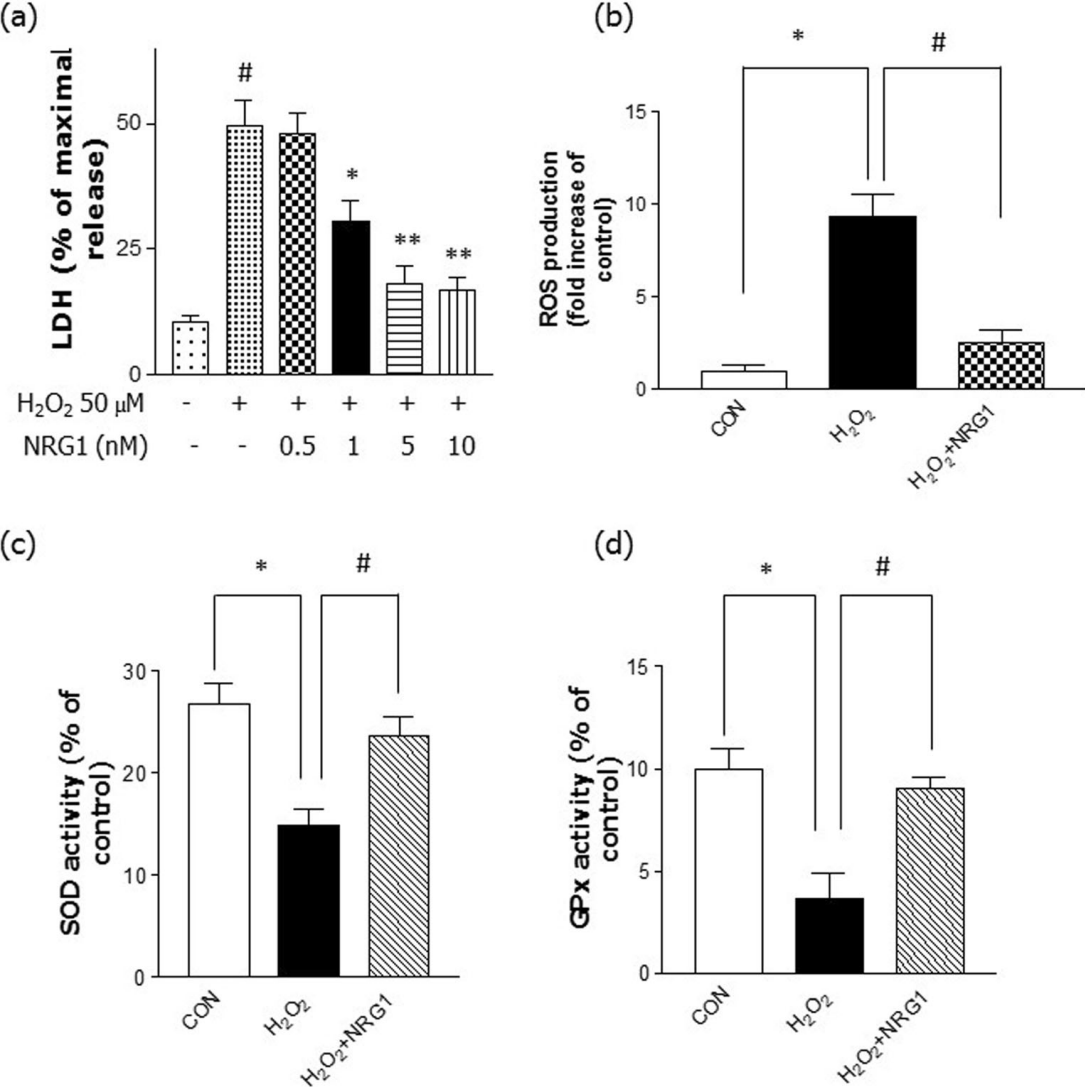
NRG1 attenuated the cell death and oxidative stress induced by H_2_O_2_. **a** NRG1 attenuated the neuronal cell death that was induced by 50 μM H_2_O_2_ treatment in primary cortical neurons. The degree of cell death was assessed for 24 h after 50 μM H_2_O_2_ treatment using LDH activity in the medium. The addition of NRG1 (0.5, 1, 5, or 10 nM) reduced the cytotoxicity induced by 50 μM H_2_O_2_ in a dose-dependent manner; *n* = 5, *P < 0.05, **P < 0.01. **b** NRG1 reduced the increase in ROS accumulation that was induced by H_2_O_2_. After 24 h, ROS was detected using fluorescence microscopy and a DCFH-DA dye that was administered to H_2_O_2_-treated primary neuronal cells in the presence or absence of 5 nM NRG1; *n* = 6, *, ^#^*P* < 0.05. **c** Primary cortical neurons were treated with 50 μM H_2_O_2_ either alone or with PBS or 5 nM NRG1 for 24 h. SOD activity was evaluated by measuring the inhibition of the reduction of tetrazolium salt by xanthine-xanthine oxidase per the manufacturer’s instructions (Cayman Chemical Company, MI, USA). The SOD assay measured all three types of SOD (Cu/An, Mn, and FeSOD). *n* = 6, *, ^#^*P* < 0.05. **d** After the primary cortical cells were exposed to 50 μM H_2_O_2_ in the presence or absence of 5 nM NRG1 for 24 h, GPx activity was measured. GPx activity was measured using a GPx assay kit (BioVision, CA, USA). *n* = 6, *, ^#^*P* < 0.05

To determine whether NRG1 affects the observed cytotoxicity in primary cortical neurons after H_2_O_2_ treatment, an LDH release assay was performed following 24 h of H_2_O_2_ treatment. The cells were incubated with different concentrations of NRG1 (0.5–10 nM) and then exposed to 50 μM H_2_O_2_ for 24 h. NRG1 attenuated the neuronal cell death induced by 50 μM H_2_O_2_ treatment in a dose-dependent manner in primary cortical neurons (Fig. 4a). Furthermore, we examined the protective effect of NRG1 against H_2_O_2_-induced ROS accumulation. We found that treatment with 50 μM H_2_O_2_ for 24 h significantly increased ROS levels (9.33 ± 0.318, *n* = 6, *P < 0.05) compared with the levels in controls (1 ± 0.29, *n* = 6). However, cotreatment with 5 nM NRG1 significantly attenuated the H_2_O_2_ treatment-induced increase in ROS accumulation (H_2_O_2_, 9.33 ± 1.20; H_2_O_2_ + NRG1, 2.50 ± 0.76, *n* = 8, ^#^*P* < 0.05) (Fig. 4b).

Several enzymes are important in the antioxidant defense system, so we evaluated antioxidant enzyme activity (SOD and GPx). Treatment with 50 μM H_2_O_2_ significantly reduced the activity of SOD (H_2_O_2_, 14.93 ± 1.56, *n* = 6, *P < 0.05) compared with that of the controls (CON, 26.77 ± 2.04, *n* = 6). Treatment with 5 nM NRG1 for 24 h attenuated the reduction in SOD activity induced by treatment with H_2_O_2_ (H_2_O_2_, 14.93 ± 1.56, *n* = 6; H_2_O_2_ + NRG1: 23.67 ± 1.86, *n* = 6; ^#^*P* < 0.05; Fig. 4c). Moreover, after the primary cortical cells were exposed to 50 μM H_2_O_2_ in the presence or absence of 5 nM NRG1 for 24 h, GPx activity was measured. We also found that after the cells were exposed to H_2_O_2_ for 24 h, there were obvious decreases in GPx activity (CON, 10.00 ± 1.00; H_2_O_2_, 3.67 ± 1.20, *n* = 8, *P < 0.05). Treatment with 5 nM NRG1 attenuated the decrease in GPx activity induced by treatment with H_2_O_2_ (H_2_O_2_, 3.67 ± 1.20, *n* = 6; H_2_O_2_ + NRG1, 9.00 ± 0.58, *n* = 6; ^#^*P* < 0.05; Fig. 4d).

### NRG1 Prevents ∆ψm Loss Induced by H_2_O_2_

**Fig. 5:**
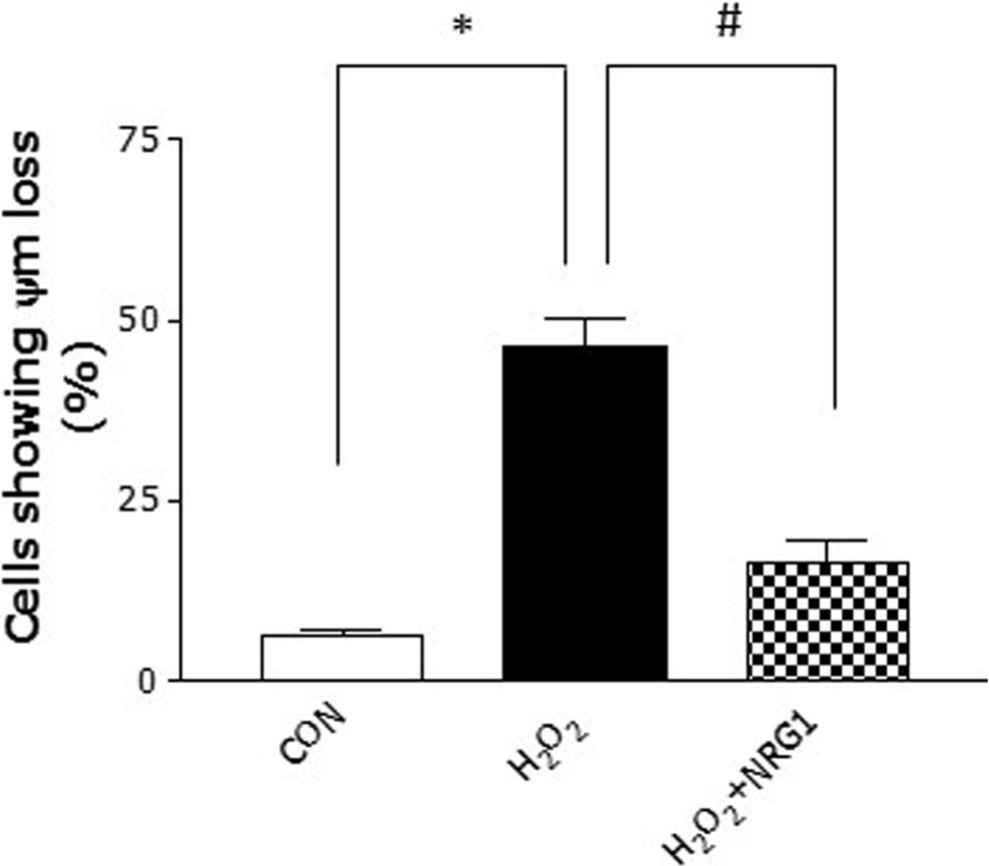
NRG1 attenuates ∆ψm loss induced by H_2_O_2_. Primary cortical neurons were treated with 50 μM H_2_O_2_ either alone or with PBS or 5 nM NRG1 for 24 h. The ∆ψm was measured with a spectrofluorometer using Rhodamine 123. *n* = 5, *, ^#^ *P* < 0.05

∆ψm has been demonstrated to play a key role in the induction of cellular death cascade by regulating the mitochondrial permeability transition pore opening (Nicolli et al. 1993). The effects of NRG1 on the regulation of ∆ψm were evaluated in primary cortical neurons after H_2_O_2_ treatment. Treatment with 50 μM H_2_O_2_ significantly reduced the activity of ∆ψm (H_2_O_2_, 46.33 ± 1.56, *n* = 5, *P < 0.05) compared with that of the controls (CON, 6.00 ± 1.15, *n* = 5). Treatment with 5 nM NRG1 for 24 h attenuated the reduction in ∆ψm induced by treatment with H_2_O_2_ (H_2_O_2_ + NRG1: 16.35 ± 3.14, *n* = 5; ^#^*P* < 0.05; Fig. 5). These results indicate that NRG1 protects neurons against the mitochondrial dysfunction induced by after H_2_O_2_ treatment.

## Discussion

The brain appears to be especially sensitive to the generation and detoxification of ROS compared with other organs. Disturbances in the balance between the generation of ROS and the antioxidative system have been reported for several neurological disorders.

In this study, we investigated whether NRG1 influences EAAC1 protein levels and oxidative stress induced by H_2_O_2_. Several previous studies have shown that exogenous H_2_O_2_ leads to oxidative stress and induces apoptotic cell death in cultured neurons (Goldshmit et al. 2001; Ratan et al. 1994). Furthermore, H_2_O_2_ inhibits uptake by recombinant rat GLRT1, GLAST, and EAAC1 reconstituted in liposomes (Trotti et al. 1996). However, it has remained unclear whether H_2_O_2_ affects the expression of EAAC1 in neurons.

Our present study shows that H_2_O_2_ reduces the expression of the EAAC1 protein. EAAC1 is expressed in neurons and is involved in cysteine uptake in the brain (Aoyama and Nakaki 2015). Cysteine transport via EAAC1 is considered key for neuronal GSH synthesis and has a unique antiapoptotic activity in neurons (De Bundel et al. 2011; Himi et al. 2003). EAAC1-deficient mice show age-dependent loss of dopaminergic neurons in the substantia nigra, which leads to the development of epilepsy due to the reduced synthesis of the neurotransmitter GABA (Berman et al. 2011). EAAC1 uptake of glutamate contributes to GABA synthesis (Mathews and Diamond 2003; Sepkuty et al. 2002). EAAC1-null mice have been found to have reduced glutathione content, increased oxidant levels, and increased susceptibility to oxidant injury (Aoyama et al. 2006; Cao et al. 2012). We have shown that H_2_O_2_ reduces the expression of EAAC1 and increases levels of cell death. Therefore, we propose that the reduction of EAAC1 induced by H_2_O_2_ may lead to or exacerbate oxidative stress, which is linked to the death of neuronal cells.

NRG1 is highly expressed in the developing brain and in the adult nervous system (Mei and Xiong 2008). NRG1 is a trophic factor whose signaling plays important roles in the maintenance of brain circuits (Li et al. 2007; Lu et al. 2014). Several lines of evidence have demonstrated that NRG1 could play a protective role in neurons against neurotoxic stimuli including ischemic insult and amyloid beta-peptide (Aβ_1-42_) (Guo et al. 2006; Woo et al. 2012). Moreover, H_2_O_2_ and Aβ_1-42_ led to a decrease in NRG1 expression in primary mouse cortical neurons (Jiang et al. 2016). H_2_O_2_- and LPS-induced neuronal toxicity down-regulated the activation of ErbB receptors and Akt1 in primary mouse cerebellar granule neuron (Xu et al. 2017).

In a previous study, we found that NRG1 exerts neuroprotective effects against the Swedish amyloid precursor protein, Aβ_1-42_, and C-terminal fragments of APP via its ErbB4 receptor (Ryu et al. 2012; Woo et al. 2012). We also showed that NRG1/ErbB4 signaling prevents the Aβ_1-42_-induced impairment of LTP (Min et al. 2011). Recently, we reported that NRG1 attenuates cognitive function impairments in a transgenic mouse model of Alzheimer’s disease (Ryu et al. 2016).

Moreover, we have reported that NRG1 induces the upregulation of EAAC1 in primary cortical neurons, resulting in an increase in glutamate uptake (Yu et al. 2015). NRG1 promotes glutathione-dependent neuronal cobalamin metabolism by stimulating cysteine uptake (Zhang et al. 2016). To investigate whether NRG1 affects the reduction of EAAC1 expression induced by H_2_O_2_, we examined EAAC1 levels via Western blotting and immunocytochemistry. We observed that NRG1 attenuated the H_2_O_2_-induced reduction in EAAC1 expression in primary cortical neurons. This result suggests that NRG1 has a protective effect on neurons.

Cells have several antioxidant mechanisms that act as a detoxifying system against ROS. SOD maintains a very low steady-state intracellular level of superoxide (Okamoto et al. 2001). H_2_O_2_ is degraded to oxygen and water by a reaction with catalase, peroxiredoxin, or GPx (Brigelius-Flohe and Maiorino 2013; Wang et al. 2007). We measured the effects of NRG1 on antioxidant enzyme (SOD and GPx) activity. NRG1 had an attenuating effect on the H_2_O_2_-induced reduction in antioxidant enzyme activity. We also found that NRG1 reduced H_2_O_2_-induced cell death and ROS production. AD patients show reduced blood antioxidant enzyme activities, including those of SOD, catalase, GPx, and GSH reductase (Casado et al. 2008). Similarly, GPx activity is significantly reduced in the substantia nigra of PD patients (Kish et al. 1985). Perturbations in cellular redox status could be closely linked to the disruption of antioxidant systems, leading to neurodegeneration.

Furthermore, we checked the effects of NRG1 on ∆ψm. The mitochondrial electron transport chain generates an electrochemical gradient through a series of redox reactions. This electrochemical gradient drives the synthesis of ATP and generates ∆ψm, which is a key indicator of cell health or injury (Ly et al. 2003; Zorova et al. 2018). In the present study, it was observed that NRG1 attenuated H_2_O_2_-induced ∆ψm. These results provide an evidence that NRG1 signaling could participate directly or indirectly in mitochondrial dysfunction.

Collectively, our results suggest that NRG1 ameliorates H_2_O_2_-induced reductions in EAACl protein levels and H_2_O_2_-induced oxidative stress. Because the current study was conducted in cultured neurons, further study is needed to clarify the effects of NRG1 on related animal disease models. More work is required to determine the activity of the transporter and the underlying regulatory mechanism of NRG1 signaling.

## Conclusion

Our results suggest that NRG1 attenuates H_2_O_2_-induced reductions in EAAC1 expression and reduces H_2_O_2_-induced oxidative stress. NRG1 may exert protective effects against oxidative stress via the regulation of EAAC1. These observations may provide novel insights into the mechanisms of NRG1 activity during oxidative stress and may reveal new therapeutic targets for regulating the oxidative stress-related effects of various neurological diseases.
